# Removal of Ammonia Using Persulfate during the Nitrate Electro-Reduction Process

**DOI:** 10.3390/ijerph19063270

**Published:** 2022-03-10

**Authors:** Shuai Yang, Xinxin Hu, Xinyu You, Wenwen Zhang, Yu Liu, Wenyan Liang

**Affiliations:** 1 Beijing Key Laboratory for Source Control Technology of Water Pollution, College of Environmental Science and Engineering, Beijing Forestry University, Beijing 100083, China; ysbjfu@163.com (S.Y.); yxybjfu@163.com (X.Y.); zhang_wen0902@163.com (W.Z.); liuyubjfu@163.com (Y.L.); 2 Water Quality Testing Center, Beijing Drainage Water Environment Development Co., Ltd., Beijing 100022, China; xinxinhu@126.com

**Keywords:** ammonia, persulfate, electro-oxidation, particle electrode

## Abstract

NH_4_^+^ is often produced during the electro-reduction of NO_3_^−^, which results in inadequate total nitrogen (TN) removal during advanced sewage treatment. In this study, the electro-reduction byproduct NH_4_^+^ was oxidized and removed using sulfate radical (SO_4_^•−^)-based advanced oxidation. Persulfate (PS) was activated by electrocatalysis, using Co/AC_0.9_-AB_0.1_ particle electrodes to produce SO_4_^•−^. Results showed that when the influent concentration of NO_3_^−^-N was 20 mg/L, a PS dosage of 5.0 mM could completely oxidize NH_4_^+^ at 0.1 A (nondetectable in effluent) reducing the TN concentration from 9.22 to 0.55 mg/L. The presence of coexisting PO_4_^3−^, CO_3_^2−^ and humic acid suppressed the oxidation and removal of NH_4_^+^. Electron spin resonance (ESR) spectra and quenching experiments revealed SO_4_^•−^ as the dominant radical in the process of indirect NH_4_^+^ oxidation, while •OH radicals only had an assisting role, and the surface accumulated free radicals were responsible for the indirect oxidation of NH_4_^+^. Cyclic voltammetry (CV) curves indicated that NO_3_^−^ was primarily reduced via atomic H*-mediated indirect reduction. Therefore, the activation of PS using Co/AC_0.9_-AB_0.1_ particle electrodes might be a promising alternative method for oxidizing byproduct NH_4_^+^ in the electro-reduction of NO_3_^−^ and reduce TN concentration in advanced sewage treatment.

## 1. Introduction

The total nitrogen (TN) content of the effluent of most sewage treatment plants can reach as high as 10 mg/L, even after primary and secondary treatment [1]. In sewage wastewater, TN is mainly composed of NO_3_^−^, with a small amount of NH_3_/NH_4_^+^ [2]. It has been well established that NO_3_^−^ and NH_4_^+^ bear a negative impact on aquatic environments, such as eutrophication and the occurrence of algal blooms [3]. Therefore, advanced treatment methods are required to reduce the TN content of sewage.

Electrochemical reduction is a feasible option for the conversion of NO_3_^−^ to N_2_, with the benefits of simple operational methods, low-environmental impact and economic efficiency [4]. The electro-reduction of NO_3_^−^ occurs via a complex process involving multi-electron transfer between the different valence states of nitrogen and its reaction products, such as N_2_H_4_, NH_3_, NH_2_OH, N_2_, N_2_O, NO, NO_2_^−^, and NO_2_ [5]. Direct reduction and indirect reduction pathways have both been shown to be involved in the electro-reduction of NO_3_^−^ [6]. During the direct reduction process, NO_3_^−^ is first adsorbed onto the electrode and then converted to NO_3_^−^_(ad)_. Electron transfer leads to the generation of NO_3_^2−^_(ad)_, NO_2_^−^_(ad)_ and other short-lived intermediates [7]. Subsequently, NO_3_^2−^_(ad)_ and NO_2_^−^_(ad)_ are converted (in a stepwise reduction process) to NO_(ad)_, then finally to N_2_ and NH_4_^+^, sometimes via the intermediates N_2_O, NH_2_OH and N_2_H_4_ [5]. The indirect reduction process refers to atomic H*-mediated reactions, where cathode surface-adsorbed atomic H* is produced via the reduction of protons. The products formed during the indirect reduction process usually include NO_2_^−^, NO, N_2_, and NH_3_/NH_4_^+^ [8]. Although NO_2_^−^ exists as the product of the rate-limiting step in both direct and indirect reduction, it can be rapidly degraded and converted to N_2_ and NH_4_^+^ [9]. Therefore, the nitrogen substances present in solution after electro-reduction mainly consist of the NH_3_/NH_4_^+^ product and residual NO_3_^−^. However, the production of NH_4_^+^ presents a problem as it is a very stable nitrogen intermediate, not only reducing the denitrification efficiency of the treatment system, but also posing a threat to the aquatic environment due to its hepatotoxicity and nephrotoxicity [10].

At present, the systems used for NO_3_^−^ electro-reduction include traditional two-dimensional (2D) anode/cathode plate systems and the newly-developed three-dimensional (3D) particle electrode bed system [7]. In both systems, the electrode material significantly influences the efficiency of NO_3_^−^ electro-reduction [11]. The electrode materials used in 2D systems usually include monometallic (Cu, Ni, Al, Pd, Pt, Pb, Ti, and Rh [12,13,14]) and bimetallic (Cu-Ni, Cu-Sn, Sn-Pd, Cu-Pd, and Cu-Zn [15,16,17,18]) catalytic electrodes. For 3D systems, the particle electrodes are composed of a carrier material loaded with a catalyst, such as Co_3_O_4_**-**TiO_2_/Ti, PdCu@OMC (OMC: ordered mesoporous carbon), Pd-Sn/AC, Cu/AC or Co/AC_0.9_**-**AB_0.1_ (AC: active carbon, AB: acetylene black) [9,19,20,21,22]. Compared to the 2D system, the addition of particle electrodes results in an increased specific surface area and availability of reactive sites, while also shortening the mass transfer distance, which enhances the removal efficiency and reaction rate [7,23]. However, accumulation of byproduct NH_4_^+^ also occurs in the 3D system [22]. In order to remove NH_4_^+^, chlorine active species are often applied, such as ClO^−^, HClO^•−^ and Cl_2_^•−^, which are generated via electrochemical reactions [24]. However, the removal efficiency highly depends on the concentration of Cl^−^, with either excessive or insufficient dosages reducing the removal effect. Moreover, Cl^−^ easily combines with organic matter and generates chlorination byproducts, such as trihalomethanes, trichloromethane and haloacetic acids, which present a secondary threat to the health of aquatic ecosystems and human populations [25]. The role of hydroxyl radicals (•OH) in the oxidization of NH_4_^+^ has also been investigated [5]. However, the rate of reaction between •OH and ammonia is slow, reducing the overall removal efficiency [26].

Persulfate (PS) oxidation technology has recently attracted increasing attention [27]. Sulfate radicals (SO_4_^•−^) are generated from the activation of PS under the influence of UV radiation, increased temperatures, magnetic fields and electrical currents [28,29]. In comparison, SO_4_^•−^ possesses a higher redox potential (2.5–3.1 V) than •OH (1.8–2.7 V), as well as a longer half-life (30–40 μs for SO_4_^•−^ and 20 ns for •OH), wider pH range (2–8 for SO_4_^•−^ and 2–4 for •OH) and a lower O−O bond breaking energy (140 kJ/mol for SO_4_^•−^ and 213.3 kJ/mol for •OH) [27,30]. As an electrophilic species, SO_4_^•−^ tends to react with alkoxy (-OR), amino (-NH_2_), and hydroxyl (-OH) groups [31]. SO_4_^•−^-based advance oxidation has been used previously in the field of landfill leachate treatment, to remove NH_4_^+^ and gaseous ammonia [32]. Therefore, in the present study, NH_4_^+^ removal was assessed using persulfate in a 3D PS-Co/AC_0.9_-AB_0.1_ system, with the aim of increasing TN removal. The effects of various operational parameters were assessed, allowing the nitrogen conversion pathway and the denitrification mechanism to be investigated. Furthermore, electrocatalytic denitrification was also carried out using Cl^−^ in a comparative system.

## 2. Materials and Methods

### 2.1. Experimental Setup

**Figure 1:**
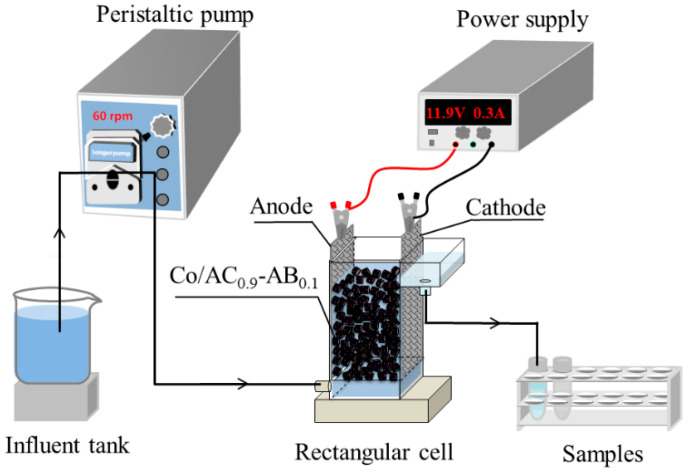
The schematic diagram for ammonia removal using electric activation of persulfate during the nitrate electro-reduction process. Co/AC_0.9_-AB_0.1_ particle electrodes are packed between a pair of anode and cathode mesh plates.

As shown in Figure 1, the mesh plates composed of Ti/RuO_2_ (12 × 2.5 cm^2^) and Ti (12 × 2.5 cm^2^) in rectangular cells were obtained from Hengli Ti Co., Ltd. (Beijing, China), for use as the anode and cathode, respectively. Then, 15 g of particle electrodes were packed between the two mesh plates (total L × W × H = 2.5 × 2.4 × 12 cm^3^). Simulated wastewater with PS dosing (namely K_2_S_2_O_8_) was continuously pumped into the reactor and treated without circulation.

### 2.2. Preparation of Particle Electrodes

The preparation of particle electrodes was performed according to a previously reported method [22]. Briefly, 90 g of powdered activated carbon (AC) and 10 g of powdered acetylene black (AB) were impregnated into 300 mL Co(NO_3_)_2_ (0.4 mol/L) and 100 mL distilled water, respectively. Then, AC and AB were separated by centrifugation and dried at 105 °C. The dried AC and AB samples were mixed thoroughly using polyvinyl alcohol as an adhesive at a mass ratio of 9:1, then molded into small cylindrical granules (~Φ × H = 4 mm × 5 mm) using a granulator. After drying at 105 °C for 24 h, the Co/AC_0.9_-AB_0.1_ particles were calcinated in a muffle furnace at 600 °C for 4 h under an N_2_ atmosphere.

### 2.3. Experimental Procedure

The simulated wastewater was prepared by dissolving KNO_3_ in distilled water (NO_3_^−^-N = 20 mg/L), using 10.0 mM Na_2_SO_4_ as an electrolyte. After 120 min, adsorption reached saturation point and the electrical current was turned on. During the study of PS dosage, PS doses were applied from 1.5–5.0 mM. The treatments were carried out under successive currents of 0.1, 0.2, 0.3, and 0.4 A for 2 h/each current. Particle electrodes were replaced with new ones after each group treatment from 0.1–0.4 A. To investigate the effects of coexisting substances, the current was set at 0.3 A and the PS dosage remained constant at 5.0 mM. The concentrations of PO_4_^3−^-P and CO_3_^2−^-C ranged from 0.5–3.0 mg/L and 50.0–200.0 mg/L, respectively. The humic acid (HA) was used to simulate the dissolved organic matter. The concentration of HA was expressed by chemical oxygen demand, namely 50.0–120.0 mg COD/L (COD) in the experiments. Therefore, the concentration of HA was expressed by COD, respectively. Particle electrodes were replaced with new ones after treatment for each coexisting substance. The hydraulic retention time (HRT) remained constant at 60 min for all experiments. To assess the comparative effect of Cl^−^, the dosage of Cl^−^ was varied from 1.5–5.0 mM, while all other conditions remained the same as described for PS experiments. Samples were collected from the outlet at 20 min intervals for the detection of NO_3_^−^-N, NO_2_^−^-N, NH_4_^+^-N, and S_2_O_8_^2−^. The experiments under initial 20 mg/L NO_3_^−^-N and without PS dosing were used as controls.

### 2.4. Identification of Reactive Species and Dominant Radicals

Free radicals were determined and recorded using an electron spin resonance (ESR) spectrometer (EMX Plus, Bruker, Karlsruhe, Germany). The parameter settings included a center field of 3513 G, seep width of 100 G, microwave power of 20 mW, and a scan time of 60 s [33]. The test samples were mixed with 50 mM 5,5-dimethyl-1-pyrrolidine N-oxide (DMPO, TCI Development, Tokyo, Japan). Quenching experiments were performed to identify the contribution of different radical species using simulated wastewater, composed of 5.0 mM PS in 6.0 mg/L NH_4_^+^-N in distilled water, using different molar ratios of [scavenger]/[PS] to screen free radicals and establish their contributions. Molar ratios of 1000:1 tert-butyl alcohol (TBA, ≥99%), 1000:1 and 2000:1 ethanol (EtOH, ≥99%) and 200:1 phenol (≥99%) were separately added to simulated sewage samples prior to treatment. Due to the potential interference caused by organic reagents, samples were filtered through a 0.22 μm membrane prior to NH_4_^+^ determination.

### 2.5. Analytical Methods

NO_3_^−^ and NO_2_^−^ were determined via ion chromatography (Dionex ICS-3000, Sunnyvale, CA, USA) using a Dionex IonPac AS11-HC analytical column (4 mm × 250 mm) and an AG11-HC guard column (4 mm × 40 mm). Elution was performed using 12.5 mM NaOH at a flow rate of 1.0 mL/min. NH_4_^+^ was analyzed using salicylic acid spectrophotometry (UV2600, Techocomp, Beijing, China) [34]. Total nitrogen (TN) was calculated as the sum of NO_3_^−^-N, NO_2_^−^-N and NH_4_^+^-N. The pH value was determined using a pH-meter (PB-10, BSISL, China). S_2_O_8_^2−^ was determined by UV spectrophotometry [35]. The activation efficiency of PS was calculated according to Equation (1), as follows:

(1) $$\mathrm{PS}\;\mathrm{activation}\;\mathrm{efficiency}\;\approx \;\frac{C_{0}-{\;C}_{t}}{C_{0}}\;\times \;100\%$$

where, *C*_0_ and *C_t_* are the PS concentration of the influent and effluent, respectively.

### 2.6. Electrochemical Measurements

Cyclic voltammetry (CV) measurements were performed using an electrochemical workstation (CHI 660D, Shanghai CH Instruments, China), with Pt filament and Ag/AgCl electrodes employed as the counter and reference electrodes, respectively. A glassy carbon disk electrode (3.0 mm) was used as the working electrode. Approximately 20.0 μL of pre-dispersed catalyst ink was coated dropwise onto the polished glassy carbon electrode. CV tests were performed with a cycle ranging from −0.39 V to +1.61 V and a scan rate of 0.1 V/s for 10 segments. A solution containing 0.5 M Na_2_SO_4_ was used as the supporting electrolyte. All solutions were prepared using ultrapure water and purged with N_2_ gas for 20 min prior to measurements. The potentials were established in reference to a reversible hydrogen electrode (RHE).

### 2.7. Statistical Analysis

The one-way analysis of variance (ANOVA) was carried out using IBM SPSS v.20.0 (SPSS Inc., Chicago, IL, USA) software. Unless otherwise stated, results were considered to indicate significant differences if *p* < 0.05.

## 3. Results and Discussion

### 3.1. Nitrogen Conversion and Removal under Different PS Dosage and Current Conditions

**Figure 2:**
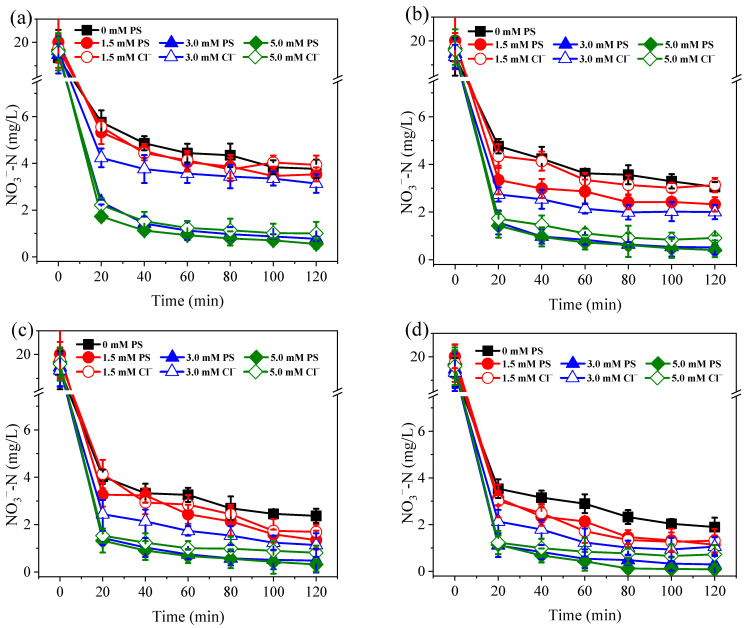
Variation of NO_3_^−^-N concentration under currents of 0.1 A (**a**), 0.2 A (**b**), 0.3 A (**c**), and 0.4 A (**d**). The dosage of PS and Cl^−^ were all set at 1.5–5.0 mM. Initial NO_3_^−^-N was 20 mg/L. The 10.0 mM Na_2_SO_4_ was used as electrolyte.

As shown in Figure 2, the Co/AC_0.9_-AB_0.1_ particle electrodes exhibited excellent catalytic activity for the electro-reduction of NO_3_^−^. For samples without PS dosing, the percentage of NO_3_^−^ conversion reached 80.5–90.2%, with the percentage conversion increasing in accordance with the applied current. This phenomenon occurred due to Co on the surface of particle electrodes having a strong catalytic activity for the production of atomic H*, which was beneficial to the indirect reduction of NO_3_^−^ [22]. Following the addition of PS at dosages of 1.5–5.0 mM, NO_3_^−^ reduction was further increased by 9.4–16.7%. Although PS activation occurred at the cathodic side of the particle electrode [36], the PS activation process did not interfere with the reduction of NO_3_^−^. PS is frequently used in advanced oxidation processes, but it can also facilitate the formation of Co-H* on electrode surfaces [36]. Therefore, PS promotes the atomic H*-mediated reduction of NO_3_^−^. Compared with the control samples, the addition of 1.5 mM PS significantly enhanced the reduction of NO_3_^−^ at 0.2–0.4 A (*p* < 0.05), although this effect was not observed at 0.1 A. When the PS dosage was increased further to 3.0 and 5.0 mM, the enhancement effect was significantly greater than that of 1.5 mM PS (*p* < 0.05). However, there were no significant differences found between 3.0 and 5.0 mM (*p* > 0.05), implying that increases in the dosage of PS beyond 3.0 mM, did not further improve NO_3_^−^ reduction.

**Figure 3:**
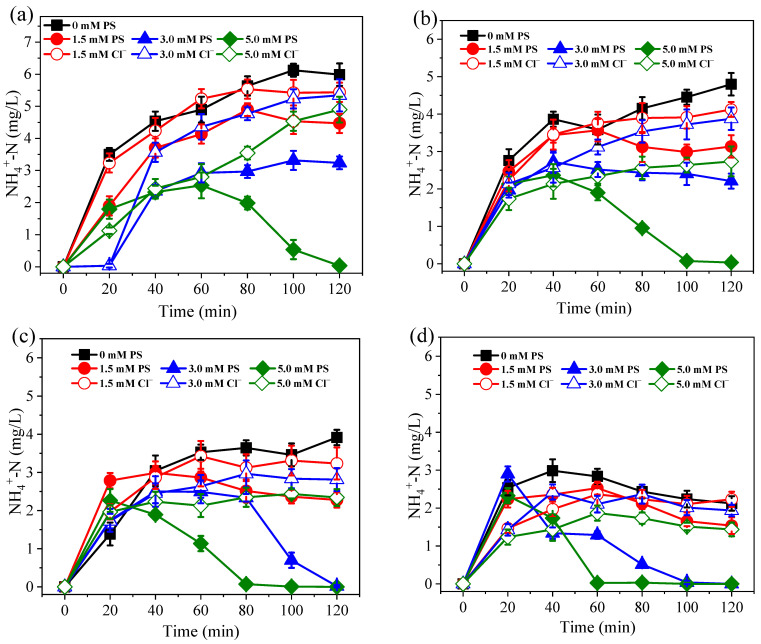
Variation of NH_4_^+^-N concentration under currents of 0.1 A (**a**), 0.2 A (**b**), 0.3 A (**c**), and 0.4 A (**d**). The dosage of PS and Cl^−^ were all set at 1.5–5.0 mM. Initial NO_3_^−^-N was 20.0 mg/L. The 10.0 mM Na_2_SO_4_ was used as electrolyte.

**Figure 4:**
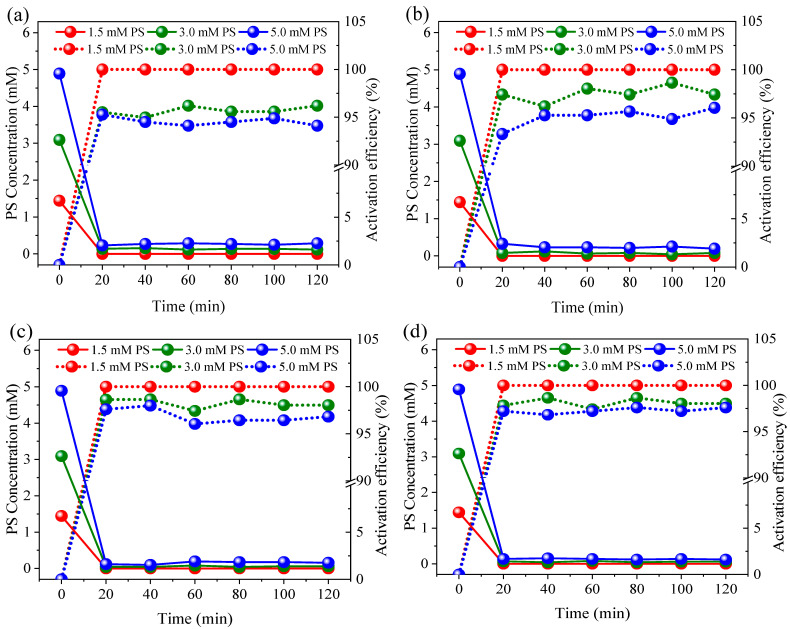
Activation efficiency of PS under currents of 0.1 A (**a**), 0.2 A (**b**), 0.3 A (**c**), and 0.4 A (**d**). Initial PS was 1.5, 3.0 and 5.0 mM. The 10.0 mM Na_2_SO_4_ was used as electrolyte.

During the electro-reduction process at 0.1–0.4 A, approximately 10.2–28.2% of NO_3_^−^ was converted into NH_4_^+^ without PS dosing (Figure 3). Therefore, when the influent NO_3_^−^-N concentration was ~20.0 mg/L, 2.1–5.5 mg/L of NH_4_^+^-N was discharged into the effluent. It is known that NH_4_^+^-N concentrations over 2.0 mg/L can cause toxicity to aquatic life, such as fish [37]. However, compared with controls, the NH_4_^+^-N concentration was significantly reduced after PS dosing at all assessed currents (*p* < 0.05). Even low current conditions of 0.1 A, and the addition of 5.0 mM PS resulted in NH_4_^+^ being undetectable in the effluent. In contrast, when 5.0 mM of Cl^−^ was added to the system, the concentration of NH_4_^+^ in the effluent continually accumulated throughout the whole experimental process, reaching 4.7 mg/L. As can be seen from Figure 4, the PS activation efficiency reached >94.1% at 0.1 A, suggesting that PS can easily be activated by Co/AC_0.9_-AB_0.1_ particle electrodes. Due to the effective activation of PS, the oxidation of NH_4_^+^ was positively correlated with PS dosage, with higher PS dosages achieving a better NH_4_^+^ oxidation efficiency. Furthermore, increases in current could overcome the effects of low PS dosage, with a current of 0.3 A resulting in NH_4_^+^ being undetectable in the effluent at a PS dose of 3.0 mM. When 3.0 mM Cl^−^ was applied, the effluent still contained 2.8 mg/L NH_4_^+^-N. Although active chlorine species have the capacity to oxidize NH_4_^+^, they cannot oxidize and remove NH_4_^+^ as efficiently as PS at an equivalent dosage.

**Figure 5:**
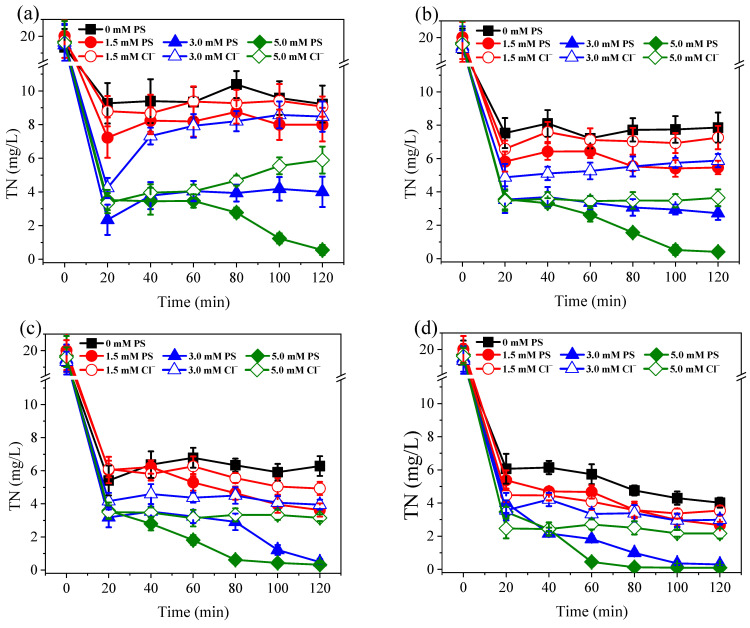
Variation of TN concentration under currents of 0.1 A (**a**), 0.2 A (**b**), 0.3 A (**c**), and 0.4 A (**d**). The dosage of PS and Cl^−^ were all set at 1.5–5.0 mM. Initial NO_3_^−^-N was 20 mg/L. The 10.0 mM Na_2_SO_4_ was used as electrolyte.

**Figure 6:**
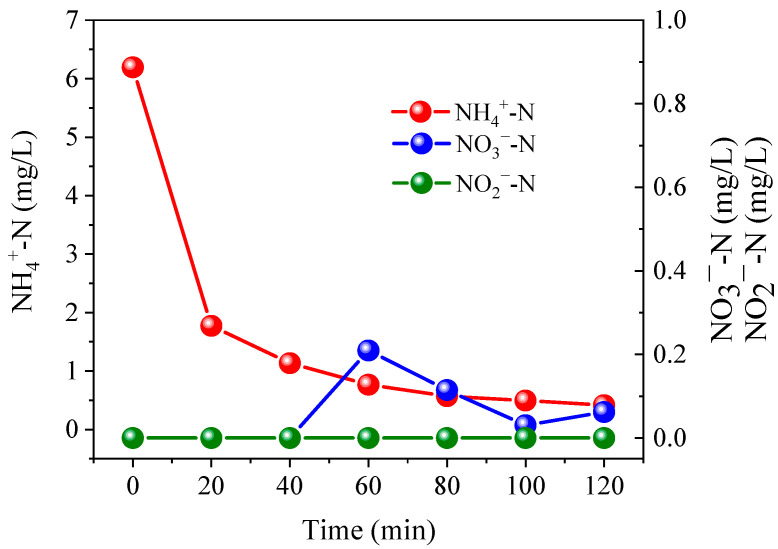
Variation of NO_3_^−^-N, NO_2_^−^-N and NH_4_^+^-N concentration under 0.3 A with 5.0 mM PS. Initial NH_4_^+^-N was 6.0 mg/L. The 10.0 mM Na_2_SO_4_ was used as electrolyte.

During the treatment processes, all samples dosed with PS achieved lower TN concentrations than the relevant controls (Figure 5). In order to investigate the NH_4_^+^ oxidation products, samples were prepared by dissolving (NH_4_)_2_SO_4_ (6.0 mg/L NH_4_^+^-N) into 10.0 mM Na_2_SO_4_, followed by treatment for 120 min at 0.3 A with the addition of 5.0 mM PS. Results showed that only 0.06 mg/L NO_3_^−^-N was generated, while NO_2_^−^-N was undetectable (Figure 6). These results suggest that the oxidation products of NH_4_^+^ mainly consisted of nitrogenous gases. The release of gaseous substances from aqueous solution resulted in a decrease in TN concentration. When a PS dosage of 5.0 mM was applied, a TN removal efficiency of >99% could be achieved, even at 0.1 and 0.2 A. In contrast, the maximum TN removal efficiency achieved with the addition of 5.0 mM Cl^−^, was only 89% at 0.4 A. Therefore, due to the high removal effects achieved under low currents, the oxidation of NH_4_^+^ by PS not only improves the denitrification efficiency of the system, but also reduces the energy consumption requirements.

### 3.2. Effects of Coexisting Substances

**Figure 7:**
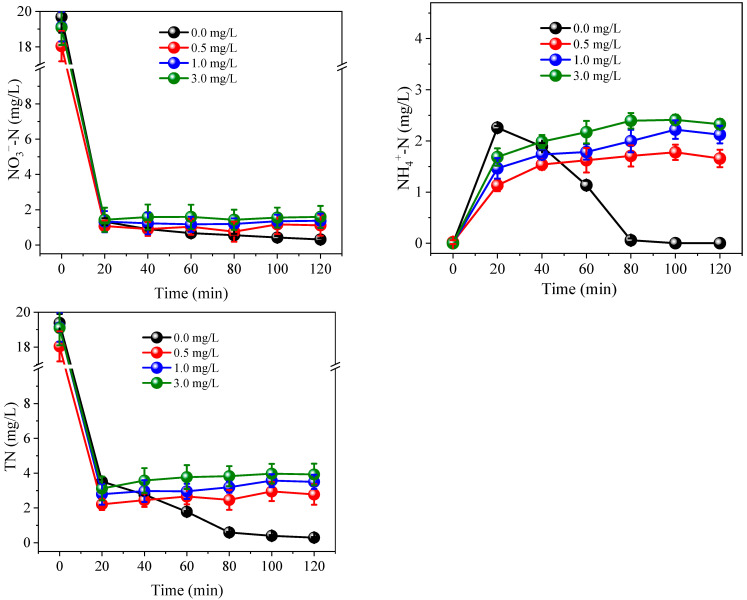
Effects of PO_4_^3−^ on the removal of NO_3_^−^-N, NH_4_^+^-N and TN. Initial NO_3_^−^-N was 20.0 mg/L. Current = 0.3 A. PS = 5.0 mM. The 10.0 mM Na_2_SO_4_ was used as electrolyte.

**Figure 8:**
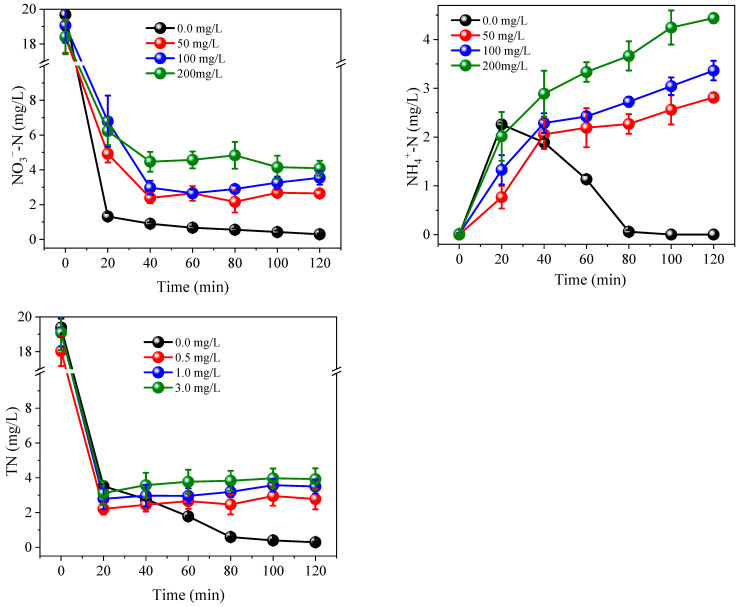
Effects of CO_3_^2−^ on the removal of NO_3_^−^-N, NH_4_^+^-N and TN. Initial NO_3_^−^-N was 20.0 mg/L, current = 0.3 A, PS = 5.0 mM. The 10.0 mM Na_2_SO_4_ was used as electrolyte.

**Figure 9:**
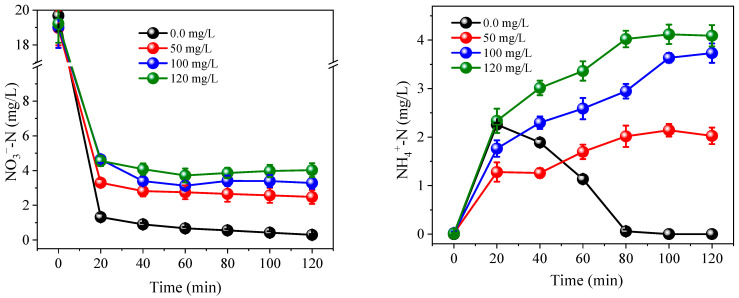

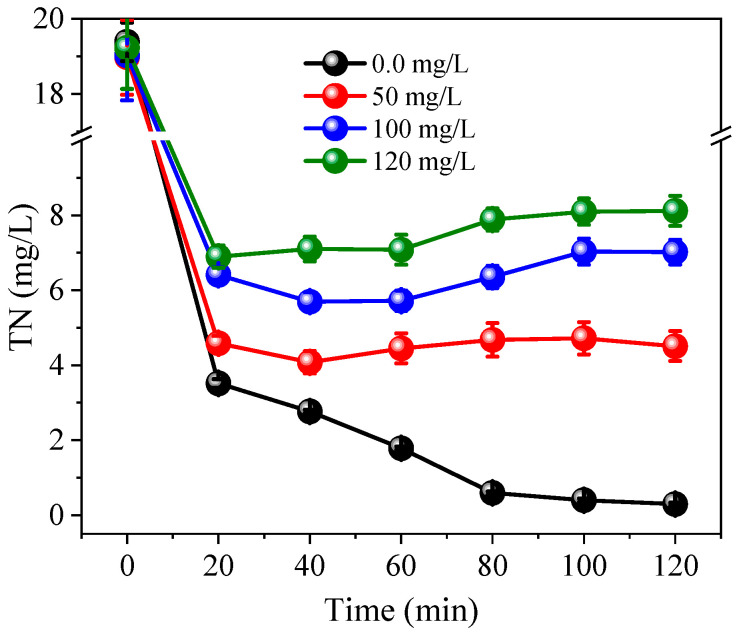
Effects of humic acid on the removal of NO_3_^−^-N, NH_4_^+^-N and TN. Initial NO_3_^−^-N was 20.0 mg/L, current = 0.3 A, PS = 5.0 mM. The 10.0 mM Na_2_SO_4_ was used as electrolyte.

Various coexisting anions and organic compounds are present in actual municipal sewage. Therefore, the effects of several commonly coexisting species, including PO_4_^3−^, CO_3_^2−^ and HA, were investigated. As shown in Figure 7, Figure 8 and Figure 9, the coexistence of PO_4_^3−^, CO_3_^2−^ and HA had a negative impact on the reduction of NO_3_^−^, especially in terms of the oxidation of NH_4_^+^. PO_4_^3−^ and CO_3_^2−^ have been shown to adsorb the surface of electrodes, resulting in competition for active sites and interfering with the adsorption and conversion of NO_3_^−^ [38,39]. HA is an amphoteric substance that can be directly or indirectly reduced on the surface of Co/AC_0.9_-AB_0.1_ particle electrodes, with both systems interfering with the reduction of NO_3_^−^ [22]. During the oxidation of NH_4_^+^, HPO_4_^2−^ (hydrolyzed by PO_4_^3−^) and CO_3_^2−^ often act as scavengers of the free radicals SO_4_^•−^ and •OH, therefore reacting with SO_4_^•−^ and •OH to yield weak oxidants, such as HPO_4_^•−^ and CO_3_^•−^ (Equations (2)–(5)) [39].

(2) $${\mathrm{SO}}_{4}^{•-}{+\mathrm{HPO}}_{4}^{2-}\;\to {\;\mathrm{SO}}_{4}^{2-}{+\mathrm{HPO}}_{4}^{•-}$$

(3) $${•\mathrm{OH}+\mathrm{HPO}}_{4}^{2-}\;\to {\;\mathrm{HPO}}_{4}^{•-}{+\mathrm{OH}}^{-}$$

(4) $${\mathrm{SO}}_{4}^{•-}{+\mathrm{CO}}_{3}^{2-}\;\to {\;\mathrm{SO}}_{4}^{2-}{+\mathrm{CO}}_{3}^{•-}$$

(5) $${•\mathrm{OH}+\mathrm{CO}}_{3}^{2-}\;\to {\;\mathrm{OH}}^{-}{+\mathrm{CO}}_{3}^{•-}$$

Similarly, HA can also be oxidized by consuming SO_4_^•−^ and •OH [36]. Therefore, the coexistence of these substances reduced the oxidation performance of the radical-based system. The oxidation of NH_4_^+^ was greatly affected and the efficiency of NO_3_^−^ and NH_4_^+^ removal was reduced, negatively affecting TN removal. However, after actual sewage treatment, the TP content of the effluent typically ranges from 0.5–1.0 mg/L, while the concentration of HA, expressed as COD, is generally less than 50.0 mg/L [40] and therefore, the concentration of NH_4_^+^-N would be below 2.0 mg/L under this coexisting substance concentration, which would not adversely affect the water environment. Even if the concentration of CO_3_^2−^-C reached 50.0 mg/L, a concentration of NH_4_^+^-N in the effluent would measure 2.8 mg/L, which would only have a slight influence on the water environment. However, the negative impact can be mitigated by increasing the PS concentration accordingly.

### 3.3. Reactive Species Identification

**Figure 10:**
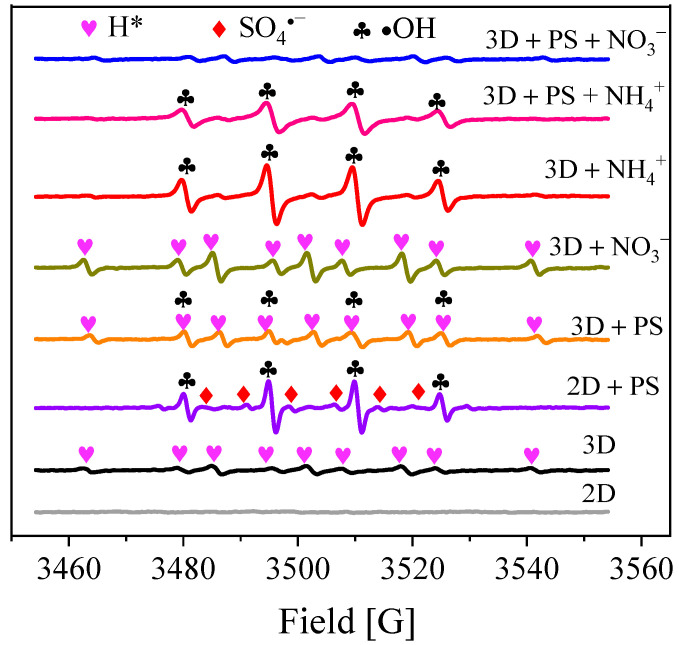
ESR spectra of electrolysis using DMPO spin-trapping under different systems. Current = 0.3 A, PS = 5.0 mM, DMPO = 50.0 mM.

In order to confirm the active radical species in the PS-Co/AC_0.9_-AB_0.1_ system, ESR experiments were carried out under different conditions. As shown in Figure 10, there were no free radical signals detected in the 2D system. After the addition of PS (2D + PS), a weak signal was observed for the DMPO-SO_4_ adduct (1:1:1:1:1:1, a_N_ = 13.2 G, a_H_ = 9.5 G, a_N_ = 1.4 G, a_H_ = 0.8 G), whereas the DMPO-OH adduct signal (1:2:2:1, a_N_ = a_H_ = 14.9 G) was much stronger. This suggests that DMPO-SO_4_ and DMPO-OH were both generated from the activation of PS, while the •OH radical was yielded from SO_4_^•−^ reacting with H_2_O/OH^−^, as shown in Equations (6) and (7) [39]:

(6) $${\mathrm{SO}}_{4}^{•-}{+\mathrm{OH}}^{-}\;\to {\;•\mathrm{OH}+\mathrm{SO}}_{4}^{2-}$$

(7) $${\mathrm{SO}}_{4}^{•-}{+\mathrm{OH}}^{-}\;\to {\;•\mathrm{OH}+\mathrm{SO}}_{4}^{2-}$$

For the 3D system, a weak signal was observed for the DMPO-H adduct (1:1:2:1:2:1:2:1:1), with hyperfine coupling constants of a_N_ = 15.5 G and a_H_ = 20.6 G. This indicated that Co/AC_0.9_-AB_0.1_ particle electrodes had performed hydro-reduction of NO_3_^−^. Atomic H* was generated by the electrolysis of H_2_O with catalytically active Co on the particle electrode [22]. After the addition of PS (3D + PS), a new adduct signal (1:2:2:2:2:2:2:2:1) was observed, which was ascribed to the original signal of DMPO-H overlapped with that of DMPO-OH [36]. However, the signal for DMPO-OH was weaker than that of the 2D + PS system, which was due to neutralization of the generated H* and •OH. Moreover, the DMPO-SO_4_ signal was too weak to be detected in the 3D + PS system, which can be ascribed to the much higher reaction rate constant between •OH and DMPO compared to between SO_4_^•−^ and DMPO [41].

As seen in Figure 10, after NO_3_^−^ was added to the 3D system (3D + NO_3_^−^), rather than disappearing, the DMPO-H signal was slightly strengthened. This implies that the generation rate and the amount of atomic H* in the 3D system was large enough to reduce NO_3_^−^. When NH_4_^+^ was added to the 3D system (3D + NH_4_^+^), the DMPO-H signal disappeared and a signal for DMPO-OH emerged. As for the 3D + PS + NH_4_^+^ system, the DMPO-OH signal weakened slightly thereafter, although it remained consistently strong. Although DMPO-OH was generated in both 3D + NH_4_^+^ and 3D + PS + NH_4_^+^ systems, the NH_4_^+^ removal efficiencies of these two systems were apparently different. NH_4_^+^ could not be effectively oxidized and removed in the 3D system, while in the 3D + PS system NH_4_^+^ was completely oxidized and removed. Although the DMPO-SO_4_ signal was not observed in the 3D + PS system, SO_4_^•−^ and •OH can interconvert, with •OH being transferred to SO_4_^•−^ via the assistance of SO_4_^2−^ and HSO_4_^−^ (Equations (8) and (9)) [31,42]. Furthermore, the slow reaction rate constant for •OH and NH_3_/NH_4_^+^ led to the low level of NH_3_/NH_4_^+^ oxidation by •OH [26,43]. Therefore, it can be inferred that the oxidation of NH_4_^+^ occurred mainly through its reaction with SO_4_^•−^ in the 3D + PS system, resulting in the NH_4_^+^-N concentration measuring below the detection limit (0.04 mg/L).

(8) $${•\mathrm{OH}+\mathrm{SO}}_{4}^{2-}\;\to {\;\mathrm{SO}}_{4}^{•-}{+\mathrm{OH}}^{-}$$

(9) $${•\mathrm{OH}+\mathrm{HSO}}_{4}^{-}\;\to {\;\mathrm{SO}}_{4}^{•-}{+H}_{2}O$$

As shown in Figure 10, nearly no free radical signals could be detected in the 3D + PS + NO_3_^−^ system. The disappearance of the DMPO-H signal indicated hydro-reduction of NO_3_^−^, while disappearance of the signals for DMPO-OH and DMPO-SO_4_ occurred as a result of consumption of NH_4_^+^ produced from the electro-reduction of NO_3_^−^. In addition, due to the reaction of SO_4_^•−^ with NH_4_^+^, more •OH was converted into SO_4_^•−^, further weakening the DMPO-OH signal.

### 3.4. Identification of Dominant Radical

**Figure 11:**
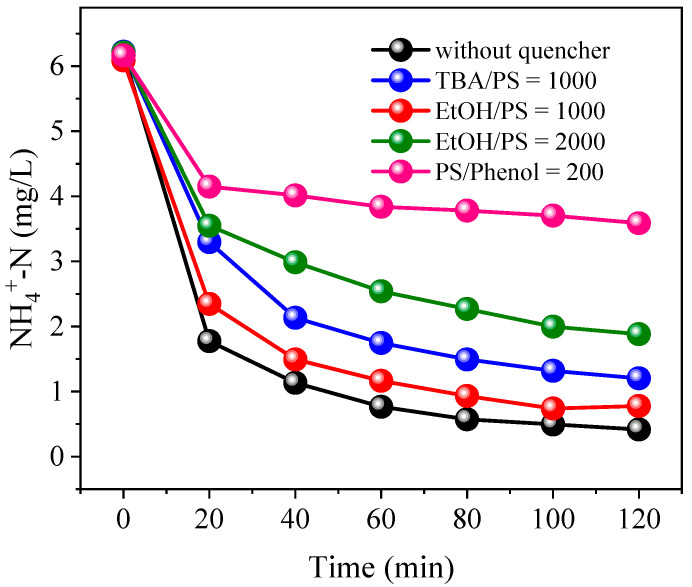
Variation of NH_4_^+^-N concentrations under PS-Co/AC_0.9_-AB_0.1_ system with different molar ratios of TBA/PS, EtOH/PS and phenol/PS. Current = 0.3 A. PS = 5.0 mM. HRT = 60 min. Initial NH_4_^+^-N = 6.0 mg/L. The 10.0 mM Na_2_SO_4_ was used as electrolyte.

In order to establish the relative contributions from SO_4_^•−^ and •OH in the oxidation of NH_4_^+^, free radical quenching experiments were conducted. TBA is typically used as a scavenger of •OH, as the second reaction rate (k) of k_•OH_ ((3.8–7.6) × 10^8^ M^−1^s^−1^) is about 1000-fold greater than that of $k_{{\mathrm{SO}}_{4}^{•-}}$ ((4.0–9.4) × 10^5^ M^−1^s^−1^) [44]. EtOH and phenol can both effectively scavenge •OH and SO_4_^•−^ with rate constants of k_•OH/EtOH_ = (1.2–2.8) × 10^9^ M^−1^s^−1^, $k_{{\mathrm{SO}}_{4}^{•-}/\mathrm{EtOH}}=$(1.6–7.7) × 10^7^ M^−1^s^−1^, k_•OH/phenol_ = 6.6 × 10^9^ M^−1^s^−1^ and $k_{{\mathrm{SO}}_{4}^{•-}/\mathrm{phenol}}=$ 8.8 × 10^9^ M^−1^s^−1^ [42,45]. As shown in Figure 11, the NH_4_^+^ removal efficiency reached 93.3% without the addition of a quenching agent. When the molar ratio of TBA/PS was 1000:1, NH_4_^+^ removal was inhibited and reduced to 80.7%, indicating •OH had participated in the oxidation of NH_4_^+^. When the molar ratio of phenol to PS was 200:1, NH_4_^+^ removal decreased sharply to 45.3%. The number of radicals necessary for TBA to quench oxidation is approximately consistent with phenol [36]. However, the inhibition of NH_4_^+^ oxidation caused by the addition of a 200:1 molar ratio of phenol was greater than that of 1000:1 molar ratio of TBA, indicating that the contribution of SO_4_^•−^ was much greater than that of •OH. Therefore, it can be concluded that SO_4_^•−^ played a dominant role in the oxidation of NH_4_^+^, while the contribution from •OH was less.

Although EtOH can effectively scavenge both SO_4_^•−^ and •OH, the addition of EtOH (molar ratio to PS of 1000:1) caused only a 6.0% reduction in NH_4_^+^ oxidation (Figure 11). Increasing the EtOH/PS molar ratio to 2000:1 increased the suppression of NH_4_^+^ oxidation to 23.6%. The varied inhibitory effect of the three quenching agents was related to their physicochemical properties and the formation sites of the free radicals [46]. Phenol, TBA and EtOH have varying dielectric constants of 9.78, 12.47 and 28.40, respectively [47]. Dielectric constants reflect the polarity of a substance. Since the polarity of a substance usually reflects its water solubility, the hydrophobic quality of the quenching agents can be ranked in the descending order of phenol > TBA > EtOH. Generally, hydrophobic quenching agents easily react with free radicals present on the surface of catalytic materials. Powder activated carbon has the property of highly selective adsorption of hydrophobic organic compounds [48]. Therefore, the hydrophobic phenol can more easily approach the particle electrode surface and react with surface-bound free radicals, while hydrophilic TBA and EtOH prefer to compete for •OH and SO_4_^•−^ in the liquid phase [47,49]. The greater inhibitory effect of phenol compared to TBA and EtOH, indicates that a majority of •OH and SO_4_^•−^ were accumulated on the surface of particle electrodes, with the free radical reaction with NH_4_^+^ mainly occurring in the boundary layer on the surface of Co/AB_0.9_-AC_0.1_.

### 3.5. Electrocatalytic Performance of PS-Co/AC_0.9_-AB_0.1_

**Figure 12:**
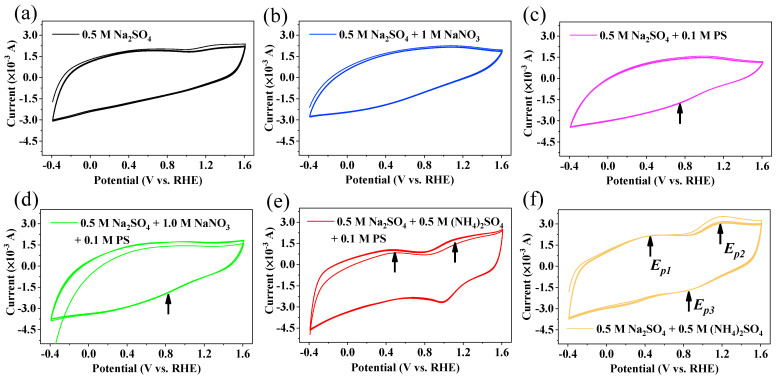
Cyclic voltammetry curves of Co/AC_0.9_−AB_0.1_ particle electrodes under different electrolyte. 0.5 M Na_2_SO_4_ (**a**), 0.5M Na_2_SO_4_ + 1 M NaNO_3_ (**b**), 0.5 M Na_2_SO_4_ + 0.1 M PS (**c**), 0.5 M Na_2_SO_4_ + 1.0 M NaNO_3_ + 0.1 M PS (**d**), 0.5 MNa_2_SO_4_ + 0.5 M (NH_4_)_2_SO_4_ + 0.1 M PS (**e**) and 0.5 M Na_2_SO_4_ + 0.5 (NH_4_)_2_SO_4_ (**f**). Scan rate = 100 mV/s. Scan for 5 cycles until stable.

The PS-Co/AC_0.9_-AB_0.1_ redox process of electrocatalytic denitrification was investigated via CV analysis, with all tests based on the Na_2_SO_4_ electrolyte. As shown in Figure 12a, only one oxidation peak was observed in the Na_2_SO_4_ electrolyte at a potential (*E_p_*) of 1.44 V, which was ascribed to the oxidation of Co^0^ to Co^2+^/Co^3+^ [50]. However, no peak was observed for the reduction of Co^2+^/Co^3+^ to Co^0^ during CV cycles, suggesting that Co^2+^/Co^3+^ might receive electrons transferred from the power source to the cathode surface through a circuit, resulting in conversion of the Co valence state [22,51]. CV curves are often used to reflect heterogeneous charge transfer from an electrode to an electroactive species, although it cannot determine charge transfer and valency changes inside the electrode [9,52]. Therefore, the conversion of Co^2+^/Co^3+^ to Co^0^ mainly serves as an electron shuttle. When NO_3_^−^ was added to the electrolyte solution, no peak of NO_3_^−^ direct reduction appeared (Figure 12b), indicating that the direct electro-reduction of NO_3_^−^ was difficult, with most NO_3_^−^ reduction occurring via a reaction with atomic H* adsorbed on the surface of the Co/AC_0.9_-AB_0.1_ electrode.

**Figure 13:**
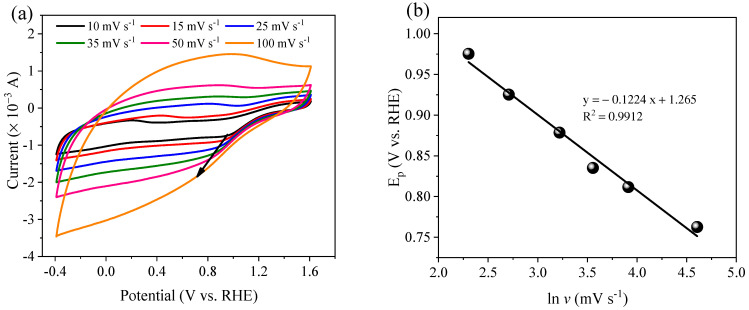
Cyclic voltammetry curves of Co/AC_0.9_−AB_0.1_ in the 0.1 M Na_2_SO_4_ + 0.1 M PS at a different scan rate (**a**), and corresponding plot of peak potentials versus the natural logarithm of scan rate in range 10–100 mV s^−1^ (**b**).

When PS was added to the electrolyte solution (Figure 12c), the current response decreased in anodic sweeps. In contrast, the current response increased in cathodic sweeps, indicating the occurrence of strong electron exchange at the electrode, with a weak reduction peak appearing at 0.76 V. Similarly, a reduction peak was observed at 0.83 V in the NaNO_3_ + PS system (Figure 12d), with the current also increasing in cathodic sweeps. These peaks were attributed to breakage of the O−O bond of PS [42,53]. The addition of PS promoted the production of atomic H* via the electrolysis of water at the cathode, resulting in an increase in electrode current. In order to establish the PS activation process, CV tests were conducted using different scan rates (*v*) (Figure 13). As demonstrated in Figure 13a,b, the reduction peak current gradually increased in accordance with the sweep rate, while *E_p_* shifted negatively. *E_p_* was found to be proportional to ln *v* (R^2^ = 0.991), indicating that PS activation was irreversible [54]. The electron transfer number (*n*) was calculated using the Laviron method (Equation (10)) [55,56]. Based on the CV test results (Figure 13), *n* value of 1.00 implies that PS activation occurred via a single electron transfer process.

(10) $$E_{p}{=E}^{o}-\left(\mathrm{RT}/\alpha nF\right)\ln(nF/{\mathrm{RTk}}_{s})\;-\;(\mathrm{RT}/\alpha nF)\ln v$$

where, *α* represents the electron transfer coefficient; *n* represents the electron transfer number; k_s_ is the diffusion coefficient; R is the gas constant (8.315 J/(K∙mol); *F* is the Faraday constant (96,500 C/mol); T is the temperature; and *v* is the sweep rate.

**Figure 14:**
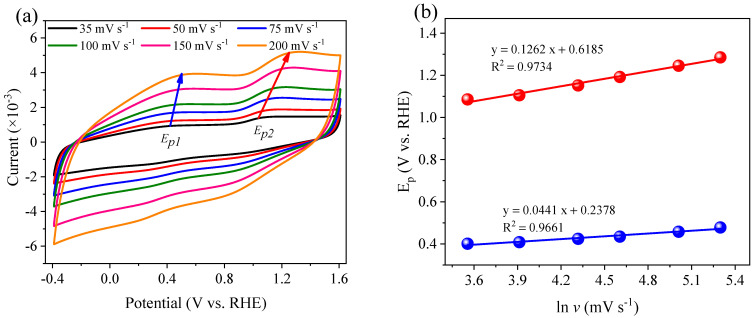
Cyclic voltammetry curves of Co/AC_0.9_-AB_0.1_ in the 0.1 M Na_2_SO_4_ + 0.5 M (NH_4_)_2_SO_4_ at a different scan rate (**a**), and corresponding plot of peak potentials versus the natural logarithm of scan rates in range 35–200 mV s^−1^ (**b**).

As shown in Figure 12f, when (NH_4_)_2_SO_4_ was added to the electrolyte solution, two oxidation peaks were observed in the anodic sweeps at 0.42 V (*E_p_*_1_) and 1.18 V (*E_p_*_2_), with electron transfer numbers of 2.86 and 1.00, respectively (Figure 14). As shown in Equations (11)–(13), *E_p_*_1_ was considered as the oxidation of NH_3_/NH_4_^+^ to N_2_, which involved a three-electron transfer reaction. When N_2_ was not rapidly separated from liquid, it was adsorbed by the electrode, causing a single-electron reduction reaction at 0.82 V (*E_p_*_3_) in the cathodic sweep and generating NH_2_OH (NH_3_OH^+^) as an intermediate (Equation (14)) [7]. NH_2_OH can also be oxidized into N_2_ via single-electron transfer (Equation (15)), resulting in the *E_p_*_2_ oxidation peak. When PS was added to the (NH_4_)_2_SO_4_ solution (Figure 12e), the peak currents *i_p_*_1_ and *i_p_*_2_ decreased from 2.18 and 3.09 mA to 1.03 and 1.74 mA, respectively. This occurred due to the generation of SO_4_^•−^ and •OH accelerating the NH_4_^+^ oxidation rate near the electrode surface, resulting in a decrease of NH_4_^+^-N absorbed on the electrode surface. However, the NH_4_^+^ in solution could not diffuse to the electrode surface fast enough, weakening the direct oxidation of NH_4_^+^ and NH_2_OH on the electrode and therefore, reducing the current response.

(11) $${\mathrm{NH}}_{3}{+H}^{+}↔{\mathrm{NH}}_{4}^{+}$$

(12) $${\mathrm{NH}}_{3}{+3\mathrm{OH}}^{-}-{\;3e}^{-}\;\to {\;1/2N}_{2}{+3H}_{2}O$$

(13) $${\mathrm{NH}}_{4}^{+}-{\;3e}^{-}\;\to {\;1/2N}_{2}{+4H}^{+}$$

(14) $${1/2N}_{2}{+H}_{2}{O+2H}^{+}{+e}^{-}\;\to {\;\mathrm{NH}}_{3}{\mathrm{OH}}^{+}$$

(15) $${\mathrm{NH}}_{2}{\mathrm{OH}+\mathrm{OH}}^{-}-{\;e}^{-}\;\to {\;1/2N}_{2}{+2H}_{2}O$$

### 3.6. The Mechanism of N Transformation

**Figure 15:**
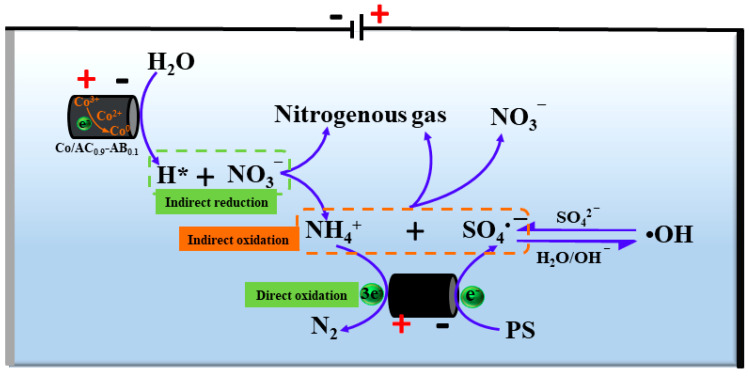
Proposed mechanism of N transformation in PS-Co/AC_0.9_-AB_0.1_ system.

On the basis of these results, a possible mechanism was proposed for NO_3_^−^ reduction and NH_4_^+^ oxidation in the PS-Co/AC_0.9_-AB_0.1_ electrocatalytic system (Figure 15). The results of CV test showed that PS activation occurred at the cathode of particle electrodes, generating SO_4_^•−^ and subsequently causing •OH to be produced from SO_4_^•−^. The process of PS activation occurred via a single electron transfer process, with an activation efficiency of >94.1%. During the electro-reduction process, in our previous study [22], we discovered that when the current was not applied, the nitrate concentration of the effluent was almost unchanged after 60 min of treatment. This implied that the adsorption did not play the main role in the removal of nitrate ions. When the current was applied, NO_3_^−^ was mainly reduced by atomic H*-mediated indirect reduction, with atomic H* produced by electrolysis of H_2_O/H^+^ via Co catalysis at the particle electrodes. The addition of PS did not interfere with the reduction of NO_3_^−^, instead promoting the generation of atomic H*, which further promoted the indirect reduction of NO_3_^−^. The NO_3_^−^ reduction products consisted of nitrogenous gas, NH_4_^+^ and residual NO_3_^−^. During the process of electro-oxidation, NH_3_/NH_4_^+^ was directly oxidized via a three-electron reaction at the electrode, generating N_2_ as a product. A portion of N_2_ was reduced to NH_2_OH, which continued to undergo single-electron oxidation. The indirect oxidation of NH_4_^+^ occurred mainly via SO_4_^•−^, while •OH functioned only in assistance. Although the DMPO-OH signal was stronger than that of DMPO-SO_4_, the slow reaction rate constant between •OH and NH_4_^+^ resulted in a poor NH_4_^+^ removal efficiency. The consumption of SO_4_^•−^ could be compensated for by the interconversion between SO_4_^•−^ and •OH. The oxidation of NH_4_^+^ occurred mainly due to radicals accumulated on the surface of particle electrodes, with the products mainly consisting of nitrogenous gas and a small amount of NO_3_^−^.

## 4. Conclusions

Oxidation of the byproduct NH_4_^+^-N can be achieved using SO_4_^•−^-based advanced oxidation under low current conditions, resulting in NH_4_^+^ being undetectable in the effluent, while also greatly reducing the TN concentration. Compared with active chlorine species, the oxidation of NH_4_^+^ via SO_4_^•−^ species can improve the denitrification efficiency and reduce energy consumption of the treatment system. SO_4_^•−^ can be efficiently and easily produced via the single-electron transfer process of PS on the cathodic side of Co/AC_0.9_-AB_0.1_. The PS activation process was found to promote the reduction of NO_3_^−^, rather than interfere with it. The direct reduction of NO_3_^−^ on the surface of Co/AC_0.9_-AB_0.1_ did not occur easily, with atomic H*-mediated indirect reduction representing the primary pathway of NO_3_^−^ reduction. However, the direct oxidation of NH_4_^+^ on the Co/AC_0.9_-AB_0.1_ surface can be achieved via a three-electron transfer process, generating N_2_ as a product. During the indirect oxidation of NH_4_^+^, SO_4_^•−^ played the dominant role while •OH acted only in assistance. Furthermore, the indirect oxidation of NH_4_^+^ was primarily accomplished by radicals that had accumulated on the surface of particle electrodes. The NH_4_^+^ oxidation products mainly consisted of nitrogenous gases, with very small amounts of NO_3_^−^-N and undetectable levels of NO_2_^−^-N present in the effluent. Therefore, the activation of PS using Co/AC_0.9_-AB_0.1_ particle electrodes might be a promising alternative method for oxidizing the byproduct NH_4_^+^ in the electro-reduction of NO_3_^−^ and reduce TN concentration in advanced sewage treatment.

## Data Availability

Data generated in this study are available upon request.
